# Phosphoinositides signaling modulates microglial actin remodeling and phagocytosis in Alzheimer’s disease

**DOI:** 10.1186/s12964-021-00715-0

**Published:** 2021-02-24

**Authors:** Smita Eknath Desale, Subashchandrabose Chinnathambi

**Affiliations:** 1 Neurobiology Group, Division of Biochemical Sciences, CSIR-National Chemical Laboratory (CSIR-NCL), Dr. Homi Bhabha Road, Pune, 411008 India; 2 Academy of Scientific and Innovative Research (AcSIR), Ghaziabad, 201002 India

**Keywords:** Phosphoinositides, PI3K signaling, Actin remodeling, Phagocytosis, Dietary fatty acids, Alzheimer’s disease

## Abstract

Alzheimer’s disease is one of the neurodegenerative diseases, characterized by the accumulation of abnormal protein deposits, which disrupts signal transduction in neurons and other glia cells. The pathological protein in neurodegenerative diseases, Tau and amyloid-β contribute to the disrupted microglial signaling pathways, actin cytoskeleton, and cellular receptor expression. The important secondary messenger lipids i.e., phosphatidylinositols are largely affected by protein deposits of amyloid-β in Alzheimer’s disease. Phosphatidylinositols are the product of different phosphatidylinositol kinases and the state of phosphorylation at D3, D4, and D5 positions of inositol ring. Phosphatidylinositol 3,4,5-triphosphate (PI 3, 4, 5-P3) involves in phagocytic cup formation, cell polarization, whereas Phosphatidylinositol 4,5-bisphosphate (PI 4, 5-P2)-mediates the process of phagosomes formation and further its fusion with early endosome.. The necessary activation of actin-binding proteins such as Rac, WAVE complex, and ARP2/3 complex for the actin polymerization in the process of phagocytosis, migration is regulated and maintained by PI 3, 4, 5-P3 and PI 4, 5-P2. The ratio and types of fatty acid intake can influence the intracellular secondary lipid messengers along with the cellular content of phaphatidylcholine and phosphatidylethanolamine. The Amyloid-β deposits and extracellular Tau seeds disrupt phosphatidylinositides level and actin cytoskeletal network that hamper microglial-signaling pathways in AD. We hypothesize that being a lipid species intracellular levels of phosphatidylinositol would be regulated by dietary fatty acids. Further we are interested to understand phosphoinositide-based signaling cascades in phagocytosis and actin remodeling.

**Video Abstract**

**Supplementary Information:**

The online version contains supplementary material available at 10.1186/s12964-021-00715-0.

## Background

### Phosphatidylinositol influence actin remodeling

The phosphorylated derivatives of phosphatidylinositols (PI) are the key secondary messengers in the cell. The phosphorylation at D3, D4 and D5 positions of the inositol ring decides the type of response and the location of the derivative inside the cell [83]. In a cellular system, phosphatidylinositol and seven different phosphoinositides have specific spacial distribution and localization, which is reversibly a change according to extracellular stimuli. Phosphoinositides are the dynamic lipid molecules; hence, their levels are maintained by a tight regulation of kinases and phosphatases. Phosphatidylinositol 3-phosphate (PI3P) are synthesized by class III PI3K (Vsp34) or class II PI3K and, the location varies at endosome/plasma membrane respectively. It is majorly involved in endosomal trafficking and localized at early endosome [41, 100]. Phosphatidylinositol 4 phosphate (PI4P) largely located at golgi complex and plasma membrane, which is synthesized by PI4K and is involved in vesicle trafficking [24]. Phosphatidylinositol 5 phosphate (PI5P) is a recently discovered lipid molecule, which possesses a wide range of functions depending upon its localization at the plasma membrane, nucleus, endo-lysosomal system, and Golgi complex [84]. PI 4,5-P2 is the oldest known phosphoinositide; apart from the small pool at plasma membrane it is available at endosomes and lysosomes. PI 4,5-P2 is largely known as a substrate for phospholipase C, which generates inositol-1,4,5-triphosphate (IP3) and diacylglycerol (DAG). The major functions of PI 4,5-P2 are to regulate important cellular processes such as endocytosis, exocytosis, iron channel and transporter activity and cytoskeletal organization. It is synthesized largely from PI4P by phosphatidylinositol 4-phosphate 5-kinase (PIP5K), smaller pool of it from PI5P by phosphatidylinositol 5-phosphate 4-kinase (PIP4K) and to some extent from PIP3 by phosphatase and tensin homolog (PTEN) enzyme [77]. PI 3,5P-2 is however localized at late endosomes and lysosomes whose level increases during oxidative stress, phagocytosis, etc. [47]. Phosphatidylinositol 3,4-bisphosphate (PI 3,4-P2) lipid molecule is present at plasma membrane and early endosomes; however, the function of which is poorly understood except its involvement in early endosome dynamics [103]. The cellular abundance of PI 3,4,5-P3 is low at plasma membrane initially; however, it regulates many cellular pathways, one of which is actin remodeling during neurite growth and dendrite morphogenesis. Its synthesis is regulated by PI3K by phosphorylating PI 4,5-P2; however, hydrolysis of PIP3 by PTEN and SH2 domain-containing inositol phosphatase (SHIP) provides important signaling molecules such as PI 4,5-P2 and PI 3,4-P2 respectively [49, 77]. Apart from known functions of calcium signaling, phosphoinositides balance the cellular pathway by binding to cellular proteins and regulate their activity. Due to their negative charge, phosphoinositides may bind to positively charged residues or protein domain (e.g., PH, FYVE, PX) for their interaction with different proteins [44, 83]. PI 4,5-P2 and PI 3,4,5-P3 are the main lipid-derivatives in the process of phagosome formation and early endosome maturation during phagocytosis [42]. The secondary lipid mediators PI 4,5-P2 and PI 3,4,5-P3 concentrate at the plasma membrane to initiate the cellular processes such as endocytosis, phagosome maturation, actin polymerization and migration [51]. Conversion between PI 4,5-P2 and PI 3,4,5-P3 is crucial to maintain various cellular signaling cascades, which even includes cell polarization, chemotaxis, and phagocytosis. Formation of PI 3,4,5-P3 is carried out by PI3K and its hydrolysis into PI 4,5-P2 or PI 3,4-P2 is mediated by PTEN or SHIP, which also decides the fate of cellular response [32]. The process of phagocytosis is tightly regulated by phosphoinositides by their spacial and temporal changes. During the initial stage of foreign particle capturing and surface binding PI 4,5-P2 concentration increases via phosphatidylinositol 4 phosphate 5-kinase (PIP5kα)-mediated metabolism, probably to induce actin polymerization for pseudopod extension. At the point of engulfment and sealing at the base of phagosome actin depolymerization occurs via decrease in PI 4,5-P2 levels via PLC-γ-mediated hydrolysis. Activation of PLC-γ is achieved in a PI 3,4,5-P3 dependent mechanism for the hydrolysis of PI 4,5-P2, which also coincides with the drop in surface charge of the cell [34, 38, 87].

**Fig. 1:**
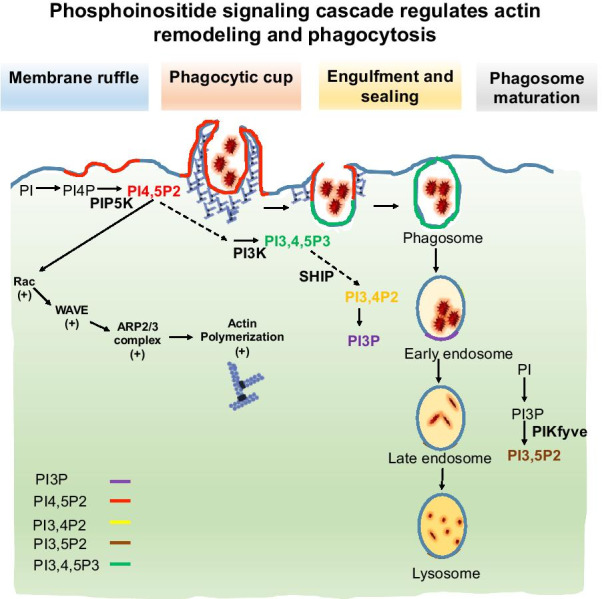
Phosphoinositide signaling cascade regulates actin remodeling and phagocytosis. In normal cells, during phagocytosis the related actin remodeling modulated by different PI species. In initial stages of phagocytosis, PI 4,5-P2 concentration increases via PIP5K, which initiates actin polymerization to extend pseudopod to catch the target. Once the target is attached to cell membrane for the process of sealing and engulfment PI 3,4, 5-P3 concentration increases via PI3K at phagosome. In the processes of phagosome maturation through early endosome, PI 3,4-P2 synthesized via SHIP and PI3P regulates trafficking. At the later stages for late endosome and lysosome mediated trafficking PI 3,5-P2 synthesized via PIKfyve. The PI 4, 5-P2 activates the ARP2/3-mediated actin polymerization via direct interaction with Rac and WASP family proteins. PI 4, 5-P2 inhibit the proteins that enhance depolymerization or inhibition of actin polymerization

The PI 4, 5-P2 regulates actin dynamics by interacting with actin-binding proteins such as ARP2/3 complex, capping proteins, WASP family proteins, and other actin-binding proteins [83]. Apart from the activation of actin-binding proteins, PI 3,4,5-P3 specifically regulates membrane ruffling via protein kinase A (PKA) (Fig. 1). PKA inhibition triggers a marked decrease in the bulk accumulation of PI 3,4, 5-P3 at membrane ruffles independent of Rac activation [28]. According to studies, the local synthesis of PI 4,5-P2 specifically by PIPKα induces actin polymerization via ARP2/3 and increases local levels of PI 3,4,5-P3 for actin remodeling, leading to membrane ruffling [35]. After ruffling, which proceeds to endocytosis and phagosome formation the concentration kinetics of PI 4,5-P2, and PI 3,4,5-P3 is mechanistically linked to required actin remodeling. PI 3,4,5-P3 concentration sharply increases at the site of phagosomal cup formation and disappears once the phagosome has been sealed off from the plasma membrane. Whereas PI 4,5-P2 levels significantly increase for circular ruffle formation and subsequently decrease during endocytosis of foreign particle. The difference in levels of PI 4,5-P2, and PI 3,4,5-P3 regulated by PI3K is mechanically important for actin remodeling and macropinosome formation [3, 42]. The negatively charged lipid such as PI 3,4,5-P3 activates N-WASP and cdc42, which triggers ARP2/3-mediated F-actin polymerization and podosomes formation. PI 3,4,5-P3 enriches membrane-associated actin regulation factors-1e (Myo1e), which links PI signaling to phagosome assembly [104]. PI 4,5-P2 synthesis by the enzyme Phosphatidylinositol-5 kinase (PI5K) from PI 4-P is trigger at the cell membrane. The overexpression of PI5K and reduced expression of phosphatase increase the levels of PI 4,5-P2, which is important for rocketing of vesicles [48]. On the other hand, the actin regulating protein binds PI 4,5-P2 with basic and hydrophobic amino acids. The interacting proteins include WASP superfamily protein, ARP2/3 complex, gelsoline family protein, and capping protein, which are affected by surface density of PI 4,5-P2. PI 4,5-P2 levels in the cell manage F-actin levels along with their association with actin polymerizing proteins, while the levels of PI 4,5-P2 depends upon the regulation of enzymes required for their synthesis [53]. However, the pool of PI 4,5-P2 in cells is largely affected by the extracellular stimulus [83]. In AD, the presence of extracellular Aβ oligomers decrease the levels of PI 4,5-P2, increases PI 3,4-P2 levels via SHIP-2 and causes hyperphosphorylation of Tau. The disrupted metabolism of PI due to Aβ affects the function of actin cytoskeleton and leads to neurotoxicity, which contributes to neurodegeneration, and synaptic failure in AD. The maintenance of the metabolism of PI has become one of the therapeutic strategies for AD [58]. Tau is another important protein in AD apart from Aβ, the hyperphosphorylation of Tau-mediated by PI3K pathway that includes GSK-3β [96]. The Aβ-induced increase in PI 3,4-P2 levels trigger Tau hyperphosphorylation in neurons. In addition, disruption of PTEN eventually increases Tau hyperphosphorylation along with decreasing PI 4,5-P2 levels [58]. The Aβ-induced increase of phospho-Tau intermediates the disease pathology and contributes to extracellular Tau seeds.

### Phosphatidylinositol 4,5-bisphosphate metabolism a therapeutic target for AD

PI 4,5-P2 is the most occupied phosphoinositide, being a substrate for phosphatidylinositol 3-kinase (PI3K), receptor activated phospholipase C and due to its signaling functions in actin remodeling and plasma membrane trafficking (Van den [99]. In the initial studies, Roberto J. Botelho et al. indicated the importance of PI 4,5-P2 as a lipid mediator which can cross the membrane and regulate the transient remodeling of actin filaments at the site of phagocytosis [9]. Scott et al., proved the importance of PI 4,5-P2 in the process of phagosome maturation and its linkage with necessary actin remodeling. Hydrolysis of PI 4,5-P2 from the site of phagocytosis is important for actin disassembly during phagosome maturation. The involvement of PI 4,5-P2 in actin remodeling indicates its pivotal role in process of rapid chemotaxis and phagocytosis where rapid actin remodeling is necessary [87]. While other groups Rohatgi et al*.*, showed the importance of N-WASP in PI 4,5-P2-mediated actin polymerization. The PI 4,5-P2 mediates the pathway of actin remodeling through N-WASP, Cdc42, and Arp2/3 complex has been proven (Fig. 1) [80].

The recent studies also suggested the importance of different PI derivatives PI3P and PI4P in the early and late stages of phagosome maturation during phagocytosis [55]. The cholesterol and sphingolipid-rich membrane rafts act as site for PI 4,5-P2 production, and membrane-associated actin polymerization via the WASP-ARP2/3 pathway [81]. PI 4,5-P2 is ideally located at inner leaflet of the plasma membrane and the occurrence of the molecule is regulated by the presence of lipid rafts and changes in membrane curvature. The PI 4,5-P2 accumulates at aggregated lipid raft regions and mediates the signaling cascade related to receptor-mediated phagocytosis [71].

**Fig. 2:**
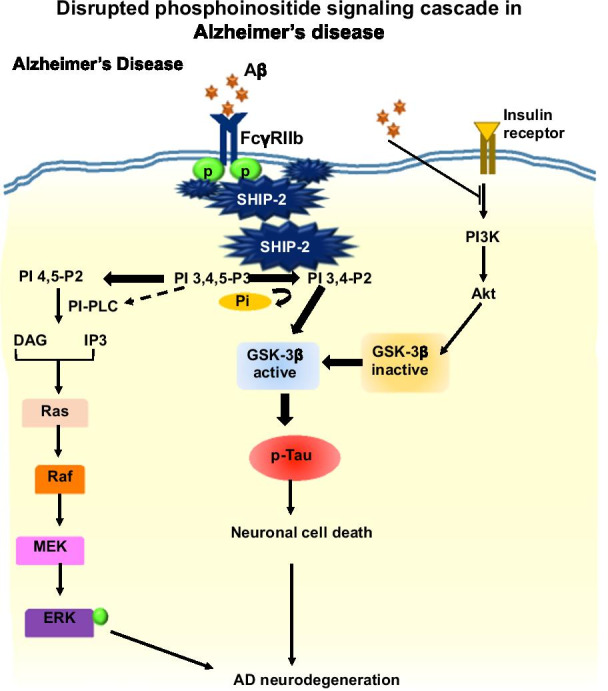
Phosphoinositides signaling cascade in Alzheimer’s disease. In AD condition, amyloid-beta known to down regulate the production of PI 4,5-P2 via SHIP-2 related mechanism. The PI 3,4,5-P3 species is increasingly hydrolyzed to PI 3,4-P2, which in turn enhances the pathway of Tau phosphorylation via GSK-3β-associated mechanism that impose neuronal death and neurodegeneration. Aβ-induced elevated concentration of PI-PLC hydrolyzes PI 4,5-P2 into IP3 and DAG which initiated Ras dependent ERK activation that eventually increases neurodegeneration. Aβ largely inhibits PI3K activity, a key player of Akt-a cell survival pathway. The reduced level of PI 4, 5-P2 down regulate the actin polymerization and phagocytosis hence there is impairment in the clearance of Aβ and increases in formation of phospho-Tau

In Alzheimer’s disease, Aβ aggregates observed to disrupt PI 4, 5-P2 metabolism (Fig. 2). The oligomeric species of Aβ-induced decrease of PI 4,5-P2, and depends upon extracellular Ca^2+^ dyshomeostasis [7]. The emerged importance of PI 4,5-P2 in neuronal survival and various signaling cascades, impose its capability as a therapeutic strategy to target in AD [4].

### Role of phosphoinositides in phagocytosis

The rapid assembly and disassembly of cortical actin cytoskeleton is important after phagosome sealing and underlying phagosome maturation [40]. F-actin assembly and disassembly decides the ability of plasma membrane curvature around the target and considerate expansion of lining membrane [10, 23]. The actin polymerization at large phagocytic cup may exhaust the determinant required for growth advancing pseudopodia; hence F-actin disassembly is important to maintain the balance during phagocytosis [69]. The polarized synthesis and spatial distribution of phosphoinositides is necessary for phagosome formation and maturation. Initially, at the time of pseudopod extensions and cell surface binding of the target PI 4,5-P2 concentration increases at the plasma membrane to induce actin polymerization. Specifically PI 4,5-P2 induce activation of WASP family proteins WAVE, WASp and mediates ARP2/3 activation to catalyze pseudopod extension [8, 80]. PI 4,5-P2 can be hydrolyzed to secondary messengers such as PI 3,4,5 P3, DAG and inositol 1,4,5 triphosphate, which are necessary for phagosome formation and maturation [61]. PI 4,5-P2-mediated actin cytoskeleton rearrangements concomitantly changes phagocytic receptors mobility, membrane traffic and even integrin activation [54]. Similarly, the PI 4,5-P2 concentration drastically decreases at the time of engulfment and sealing, which is necessary for actin disassembly. PI 4,5-P2 is necessary for the proper coating and formation of endocytic vesicles [11]. The synthesis and concentration gradient of PI 4,5-P2 is maintained by PIP5K kinase isoforms (α, β, and γ). On the other hand, the PI 3,4,5-P3 increases at the base of phagosome to maintain pseudopods around the particle. The increased synthesis of PI 3,4,5-P3 is important as it can induce PLCγ-mediated hydrolysis of PI 4,5-P [19]. The concentration of membrane associated PIP5K is necessary to maintain the PI 4,5-P2 levels at the base of phagosome and subsequent actin depolymerization for engulfment process [38]. The overall concentration of PIP5K at the plasma membrane is regulated by Rho family GTPases such as Rac and cdc42 during the process of phagocytosis. GTPase executes the highly complex actin depolymerization process occurs during phagocytosis; where, Rac is activated importantly at the base of phagocytic cup and Cdc42 at the pseudopodia region for membrane extensions [86].

### Phosphatidylinositol signaling in microglial migration

**Fig. 3:**
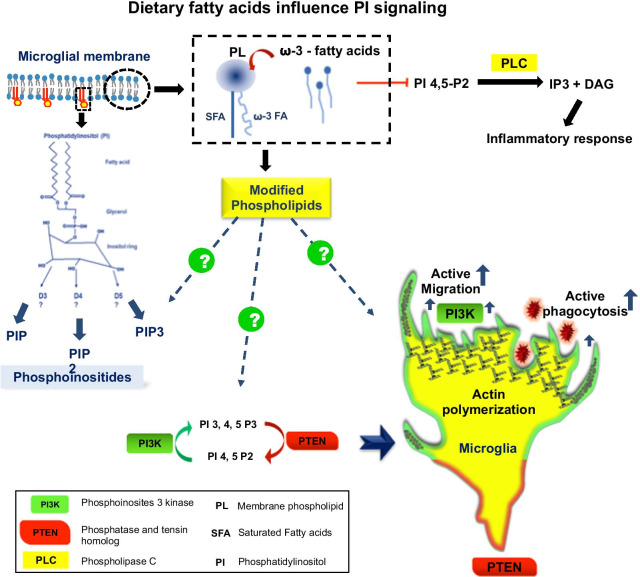
Dietary fatty acids influence PI signaling. The incorporation of dietary supplement of omega-3 fatty acids increases the potency to intercalate with the membrane glycerophospholipids. The increased omega-3 fatty acids in glycerophospholipids suspected to influence Phosphoinositides. Upon modified phospholipid content of the cell it is suspected to affect various signaling cascades during their involvement. Under physiological conditions fatty acids influence the type of PI species produced depending upon the phosphorylation at D3, D4, and D5 of inositol ring. PIP2 (PI 4, 5-P2), PIP3 (PI 3, 4, 5-P3) synthesis is maintained by the interplay of PI3K and PTEN local concentration. The highly polarized lamelliopodia-bearing cell migrates with the high concentration of PIP3 at leading ends due to local concentration gradient of PI3K. The PTEN maintains directionality and retraction at rear end via inducing higher concentration of PIP2 and lower concentration of PIP3. The positive interplay between PIP3 and PIP2 would induce active phagocytosis and migration, which is supported by actin polymerization in an activated cell. Polyunsaturated omega-3 fatty acids are suspected to induce the phagocytosis via PI signaling. Omega-3 dietary fatty acids also inhibit PLC mediated hydrolysis of PI 4,5-P2 into inositol 1, 4, 5 triphosphate (IP3) and diacylglycerol (DAG) which eventually initiates inflammatory response by microglia

Microglia is an immune cell of the brain that has surveillant nature, which is supported by high migration rates and the capacity to respond to chemotactic factors [39]. The basic actin cytoskeleton is necessary to regulate the processes such as migration and surveillant nature of microglia [26, 31]. In AD, the accumulated abnormal proteins serve in the classical activation of microglia by inducing pro-inflammatory response. The excessive pro-inflammatory response triggers neuroinflammation, which imparts the anti-inflammatory stage of microglia [26, 30]. The plasma membrane and the underlined cortical actin networks are very important for migration and phagocytosis. For the process of migration, coordinated polymerization of actin filaments provides a protrusive force (lamellipodia) and thin filamentous protrusion to sense and direct the migration (filopodia). The lamellipodia-dependent migration is carried out by actin-rich protrusion at leading ends. Whereas, filopodia sever as antennae of the cell, which probe the environment and serve pioneer in migration [66]. Lamellipodia on the other hand is formed due to coordinated actin polymerization carried by ARP2/3 complex activation [31]. The actin polymerization beneath the plasma membrane produces the protrusion that drives forward the cell at the leading end [60]. The membrane protrusion around the target for phagocytosis involves actin cytoskeleton regulation. Phosphoinositides induce migration by lamellipodia-dependent mechanism via inducing actin polymerization at leading ends and also provide directional clues during chemotaxis. Phosphatidylinositides regulate signaling by directly binding to actin-binding proteins and influence their activity [83]. The polarized gradient of PI 3,4,5-P3 after activation of chemoattractant receptor induces actin polymerization for lamellipodia-mediated migration. PI 3,4,5-P3 accumulates at the chemoattractant end and influences actin rearrangement. The gradient of PI 3,4,5-P3 is overproduced by PI3K at front end; and hydrolysis by phosphatases and tensin homology (PTEN) at retracting ends is maintained to keep the cell on track. PI 3,4,5-P3 levels polarize cell by recruiting Rac1, and DOCK2 indeed is necessary to activate ARP2/3-mediated actin filament polymerization to induce migration towards chemoattractant. The hydrolysis of PI 3,4,5-P3 to form a gradient towards chemoattractant produces high levels of PI 4,5-P2 at the uropod ends, and induce actin filament assembly to address the movement of the cell. The spatial localization of PI3K and PTEN determines the membrane localization of PI 3,4,5-P3 which creates an intracellular signaling gradient for chemotaxis [14]. The chemotactic receptors P2X, P2X4R, and P2Y12R also showed activation of the PI3K pathway over stimulation by ATP/ADP and also by Aβ [27, 39]. PI derivatives hence regulate the migration of the cells, which is necessary to engulf targets during phagocytosis (Fig. 3).

## Alzheimer’s disease pathology

Alzheimer’s diseases being a neurodegenerative disorder indicates symptoms of cognitive decline, memory loss, and finally dementia over the advancing age. The extracellular senile plaques of amyloid-beta (Aβ) and intracellular neurofibrillary tangles of Tau (NFTs) are the hallmarks of AD along with the neuroinflammation owing to activated glial network. The abnormal processing of Aβ by β-secretase produce amyloid peptide of various lengths, Aβ 40, and Aβ 42 found to accumulate in the brain [15]. However, different post-translational modifications of Tau protein detaches it from microtubule and triggers its aggregation intracellular NFTs. Tau protein released from the neuronal cells acts as a seed to introduce NFTs formation in neighboring neurons. Hence, the Tau seed behaves like “prion” and is transmitted via, synaptic or vesicular transportation [29, 90]. Microglia on other hand intervene with the Tau propagation mechanism by mediating Tau secretion via, exosomes [26, 30]. The activated microglia exacerbates Tau pathology by damaging dendrites and axons. Recently, it has been known that Tau seeds can activate NLRP3-ASC inflammasome, a multi-protein complex that recruits pro-caspase-1 via ASC to cleave proinflammatory cytokine precursors and other signaling pathways involved in immune activation of microglia [91]. Tau seeds especially oligomers have been shown to modulate the actin cytoskeleton contributes to the fact that extracellular Tau has a detrimental effect on various microglial signaling cascades [26]. Hence the presence of extracellular senile plaques of Aβ, NFTs, and Tau seeds have a consequence on neuronal networks, signal transduction in neuro-glial cells and neuroinflammation. As a comparison to Aβ oligomers, Tau aggregates and oligomers are considered as neurotoxic [75]. The presence of excessive abnormal protein and accumulation of the activated glial cell imparts neuroinflammation. In this scenario, the hampered fundamental nature of glial cells to clear the pathoproteins, contributes to neuroinflammation [85]. Production of cytokines, chemokines, and reactive oxygen species by immune cells of CNS and their duration decides the course of action or damage to the CNS. The activated microglia specifically alters transcriptional profile, produces cytokines, and undergo actin rearrangement, which differentiates the pattern of receptors expressed on the cell surface. In the severe neuroinflammatory condition, the inflammatory response overpowers the repair mechanisms carried by microglia cells. In AD, the hyper-activation of microglia and elevated production of IL-1β, TNF-α, and IL-6 contribute to neuronal synapse loss, Aβ plaque deposition, and Tau hyperphosphorylation [33].

### Aβ and Tau hampers phosphatidylinositol signaling

The amyloidogenic processing of amyloid precursor protein (APP) leads to the formation of insoluble monomer, dimer, oligomer, and aggregates of the amyloid peptide. The accumulation of amyloid plaques disrupts the neurotransmission, affects Tau pathology, and even contributes to excessive activation of glial cells. The insoluble amyloid oligomers were found to be more neurotoxic to disrupt intracellular signaling [82]. Aβ binds to various cellular receptors to induce neurotoxicity via mitochondrial dysfunction and oxidative stress leading to excessive calcium influx that instigates toxicity [13]. The soluble Aβ can interact with various receptors to activate downstream signaling pathways that produce reactive oxygen species, hyperphosphorylated Tau and also induce an inflammatory response in the brain [15]. The phosphatidylinositides metabolism is necessary for various intracellular signaling, which is affected by Aβ oligomer by activating SHIP2 via FcγRIIb receptor. Aβ aggregates have been observed to disrupt kinases, which are required to maintain PI levels in the cell. Membrane-associated phosphatidylinositol-4 kinase, phosphatidylinositol-3 kinase, phosphatidylinositol 4 phosphate kinase, and PI specific phospholipase-C were found to be disrupted by Aβ aggregates [94, 101]. PI3P synthesize by Vsp34 kinase has a fundamental role in endosomal membrane trafficking. PI3P deficiency correlates with endosome enlargement, disruption in APP processing, Aβ accumulation and defective intracellular aggregates clearance in AD [70]. In AD, excess APP reduces its ability to interact with PIKfyve complex function, a key kinase in production of PI 3,5-P2 and disrupt endosomal sorting and homeostasis [22]. The levels of several key phosphoinositides have been disrupted, which are involved in cellular processes such as phagocytosis, migration, and actin cytoskeleton remodeling. The Aβ-affected altered metabolism of phosphoinositides challenges Tau hyperphosphorylation by various protein kinases [58]. Aβ is capable of disrupting the function of phosphatidylinositol-3 kinase (PI3K) an important enzyme in the conversion of Phosphatidylinositol 4, 5-diphosphate (PI 4,5-P2) to phosphatidylinositol 3 4,5-triphosphate (PI 3,4,5-P3) and is involved in Akt-mTOR signaling pathway [17]. In AD, cholinergic agonist induced signal transduction carried out via phosphoinositide has been found to be hampered in cellular system. The deficits in GPCR mediated hydrolysis of phosphoinositide speculated to have greater impact on APP processing [57]. Phosphoinositide-3 kinase along with Ras-dependent MAPK has been reported to be evaluative in nicotine acetylcholine receptor (nAChR) functioning, which elucidates the role of PI3K in AD-related studies [106]. PI3K activation can profoundly reduce Aβ-mediated toxicity and synapse loss in the brain environment [5]. The soluble Aβ oligomer affects PI3K/Akt/GSK-3β pathway majorly causing neuronal death and Tau hyperphosphorylation [50, 56]. The disrupted PI3K signaling targets Tau hyperphosphorylation, which impart pathological condition in AD. Aggregated form of Aβ 25–35 significantly impairs phosphatidylinositol related enzyme, phospholipase C found in the brain during AD [95]. Apart from the enzymes involved in phosphatidylinositol signaling (PI), Aβ directly reduces levels of PI 4,5-P2 phospholipid that regulate various neuronal functions [7]. PI 4,5-P2 has been recognized as a vital-mediator for Aβ induced response in neurons and other brain cells. Aβ-mediated inhibition of PI3K provides PI 4,5-P2 as a substrate to PI-PLC (Phosphoinositide-phospholipase C) activity, which further activates ERK1/2 response. PI 4,5-P2 is observed to be a link between signaling pathways (PI3K/Akt and PI-PLC/ERK1/2), which has the potential to decide fate of the cell [98] (Fig. 2).

## Clinical relevance of phosphoinositides signaling

**Fig. 4:**
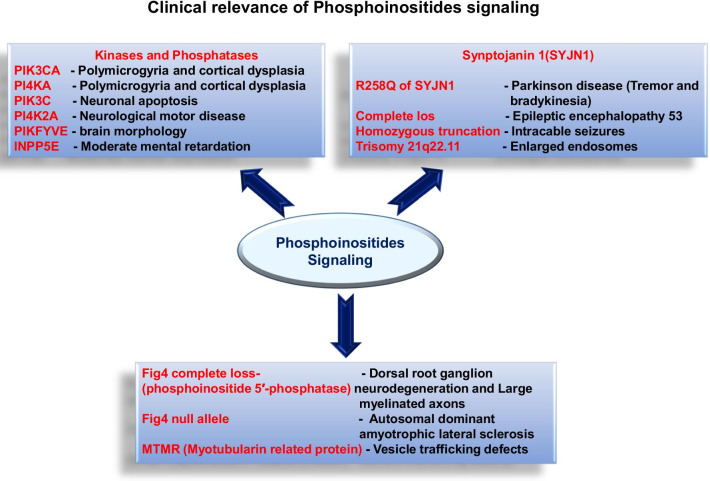
Clinical relevance of Phosphoinositides signaling. Different signaling molecules from the phosphoinositides pathway can undergo genetic mutations, which lead to various neurodegenerative diseases. The Kinases and phosphatases from the pathway and the corresponding diseases related to their mutation have been mentioned. Specifically Synptojanin 1 (SYNJ1) and in this figure genes-associated with the phosphatases are known to cause varied neurodegerative conditions, which is also link with Down’s syndrome

The ultimate target of phosphoinositides signaling is to induce an influx of Ca^2+^ via the production of DAG and inositol 1,4,5-triphosphate (IP3), activation of cellular proteins and regulate their activity. The expression analysis of PI signaling genes and their spatial distributions suggest that there is a significant abundance in glial cells and neurons. The mutations in PI kinases such as PIK3CA and PI4KA result in polymicrogyria and cortical dysplasia, which alters cerebral cortex morphology and the cellular composition. Similarly, the mutation in PIK3C3 is associated with neuronal apoptosis and neurodegeneration; whereas, loss of PI4K2A is associated with neurological motor disorder related to cerebellar and spinal cord degeneration [89, 105]. The loss of PIKFYVE, an important gene in astrocytes results in abnormal brain morphology and reduced brain weight [107]. Synaptojanin1, is a phosphoinositide phosphatase that dephosphorylate PI 4,5-P2 and PI 3,4,5-P3, which expresses predominately at nerve terminals [67]. The mutation SYJN1 likely to hamper PI 4,5-P2 at the synapses that alters synaptic vesicle cycle and contributes to the brain phenotype of the patients. Homozygous missense mutation at R258Q of SYJN1 is associated with the early onset of Parkinson disease; patients show tremor and bradykinesia with mild cerebral cortical atrophy [76]. Whereas, the complete loss of SYJN1 function is associated with early onset of epileptic encephalopathy 53, characterized by the occurrence of epileptic seizures, spastic quadriplegia and severe intellectual disability in patients [1, 46]. A homozygous truncating mutation in SYNJ1 found in a patient with intractable seizures, formation of neurofibrillary tangles and occurrence of Tau in substantial nigra of the brain [37]. Mutation at trisomy 21q22.11 consisting of SYNJ1 found in patients with Down syndrome, the disorder is indicated with enlarged endosomes due to overexpression of SYNJ1 [21]. Patients with Down syndrome have the tendency to show early onset of Alzheimer’s disease, which indicate the overexpression of APP and SYNJ1 and subsequent decrease in PI 4,5-P2 levels. Figure 4 is a phosphoinositide 5 phosphatase, which dephosphorylates PI 3,5-P2 to PI3P. The mutation in Fig. 4 gene corresponds to enlarged late endosome and lysosome along with neurodegeneration of dorsal root ganglion cells and large myelinated axons. The mouse model of the gene mutation indicates tremor and impaired motor coordination similar to Charcot-Marie-Tooth disorder in humans [18]. Myotubularin related protein (*MTMR*) mutation likely to affect vesicle trafficking defects in neurons and Schwann cell along with Fig. 4, since both control the levels of PI3,5-P2 in the cells [12, 79]. Mutations in 5′-phosphatase inositol polyphosphate 5-phosphatase E (*INPP5E*) is associated with truncal obesity, retinal dystrophy, mild mental retardation and micropenis (MORM) syndrome [45] (Fig. 4).

The signaling cascades between neurons and glia cells are regulated tightly owing to the fact that the cells are more sensitive and susceptible to death. The signaling cascades related to membrane trafficking, protein turnover, actin remodeling, clearance of accumulated proteins are tightly regulated via PI levels, interactions and concentration gradient. The association of various gene mutants from phosphoinositide pathway with neurodegenerative diseases indicate the necessity as a new therapeutic strategy.

### Dietary fatty acids govern phosphatidylinositides signaling

The preliminary fatty acids Docosahexaenoic acid (DHA, 22:3n-6) and Arachidonic acid (ARA, 20:6n-4) are the major lipids found in the brain. The n-3 and n-6 PUFA constitute the phospholipid content of the brain cell membrane [6]. DHA and ARA are important for cell signaling via varied lipid mediators. The balance between n-3/n-6 PUFA in a diet is crucially important for health and development of CNS. It has also been proved that n-3 deficiency would affect the microglial phenotype and motility required for the surveillance of CNS [64, 78]. Maternal deficiency of n-3 PUFA diet causes impairment in microglial homeostasis and phagocytic ability, synaptic pruning and subsequent behavioral abnormalities in infants [63]. The westernization of diet not only reduces n-3 fatty acids consumption but also increase saturated fatty acids intake, which has inflammatory effect on microglia [93]. Hence, balance between n-3/n-6 PUFA supplements is necessary to maintain the microglial functions. Omega-3 fatty acids especially DHA and EPA known to reduce inflammatory signaling cascades such as NF-κB, MAPK and initiate activation of anti-inflammatory factors PPAR, G-protein coupled receptor 120 (GPR120), retinoid X receptor (RXR) etc., [2, 16, 20]. N-3 PUFAs are well known to increase phagocytic ability of microglia against Aβ and myelin debris. Further the ratio between n-3/n-6 PUFA can influence the phagocytic ability of microglia depending upon the production of lipid mediators [59]. Owing to the importance of n-3 PUFA in anti-inflammatory response of microglia, it is important to understand the effect of PUFA on related signaling pathways.

In phosphatidylinositol molecule glycerol backbone is attached to inositol ring in which phosphate present at sn-3 position and two esterified acyl chains at sn-1 and sn-2 positions [36]. The acyl chain composition even changes with types of tissues, but majorly sn-1 and sn-2 are composed of stearoyl and arachidonoyl respectively [52]. Acyl chain composition in PI species is assigned via two enzymatic processes e.g. PI cycle and Land’s cycle. The Land’s cycle involves acyl chain remodeling of PI species through reactions [52]. Land’s cycle may result in specific enrichment or removal of acyl chain via action of acyltransferases and phospholipases. Remodeling imparts specific acyl chain composition to certain lipids, where Lysophosphatidylinositol acyltransferases transfer acyl chain at sn-2 position [65]. Phospholipid could act as a precursor for many bioactive lipids via action of phospholipases. Quantity and types of bioactive lipids depends upon acyl chain composition, in particular arachidonoyl and docosahexanoyl chains, which decide the downstream signaling [25]. Phosphatidylinositol-4-phosphate 5-kinase (PI4P5K), which produces PI 4,5-P2 from PI4P has important role in PI cycle. PI 4,5-P2 is well known for important physiological functions and deregulation of which has been observed in AD. PI4P5K, a rate limiting enzymes, have been observed to have acyl chain specificity of fatty acids depending upon its isoforms as a lipid activator [62, 88]. Acyl chain composition of any lipid molecule decides its function and even trigger disease state as well. Arachidonic acid is a precursor of various inflammatory molecules, which is released from the phospholipid molecule via action of phospholipase enzymes; especially phospholipase C in case of phosphatidylinositols [97]. PLC has been proved to carry out hydrolysis of inositol phosphate and produce IP3 and DAG, which are secondary lipid molecules. Free fatty acid AA has a great potential to activate PLC in an allosteric manner, which alleviate PI hydrolysis. Involvement of FFA in activating the enzyme suggests that the lipid environment of cell membrane is not inert, which may mediate the intracellular signaling [72]. Dietary fatty acids are known to induce changes in cell membrane compositions and structure, which has various effects on signal transduction pathways. Dietary fatty acids influence phosphatidylethanolamine (PE), phaphatidylcholine (PC) of the cell membrane to the most and to some extent phosphatidylinositols (PI) [102]. The omega-3 fatty acids supplement of Docosahexaenoic acid (DHA) and Eicosapentaenoic acid (EPA) specifically influence species and levels of PI in the cell, which could be considered as one of the important mechanism to regulate signaling pathways and avoid cardiac arrhythmias [73]. Omega-3 fatty acids found to inhibit the phospholipase C (PLC)-mediated hydrolysis of PI 4, 5-P2. The hydrolyzed product inositol 1, 4, 5-triphosphate (IP3) along with diacylglycerol (DAG) have been found to induces leukotriene (LTB4)-mediated inflammatory response in neutrophils [92]. The activity of PI 3, 4, 5-P3 depends upon types of fatty acids at sn-1 and sn-2 positions of phospholipids [43]. The compositions of fatty acids at sn-1 or sn-2 positions are determined by dietary fatty acids hence dietary fatty acids could influence phosphatidylinositols pool in cell. The PUFA (Polyunsaturated fatty acid) treatment to cell significantly increases the PI species. The replaced PI species by the PUFA treatment found to inhibit tumor growth by suppressing the Akt pathway. Omega-3 fatty acids tend to incorporate at the sn-2 position of glycerol backbone, that holds the tendency to change the species of phospholipid [43]. The acyl chain remodeling carried out by different enzymes might act as a mechanism that decides the particular PUFA chain at the sn-2 position of PI and determines the downstream lipid signaling molecule [25]. Different fatty acids found to influence pool specific PI derivatives in the cell. Incorporation of fatty acids into cells has been found to increase particular PI derivatives. The disrupted metabolism of PI and related signaling cascade in AD could be monitored with dietary fatty acids sources.

## Conclusion

Alzheimer’s disease is the most common cause of dementia that drags serious attention to the therapy. The two main pathological proteins are the extracellular Aβ plaques and intracellular neurofibrillary tangles of Tau. Apart from pathoproteins, neuroinflammation fabricated due to microglia after aberrant activation contributes to the disease condition and propagation. The inability of microglia to express anti-inflammatory response is one of the biggest challenges faced in the later stages of the disease. One of the approaches to design therapeutic strategy could be the induction of the anti-inflammatory nature of microglia to overcome neuroinflammation and its side effects. Fatty acids are one of the major dietary factors, which influence microglial response. Dietary fatty acids influence anti-inflammatory response by microglia is well established but it is yet to understand the important signaling molecules affected during the pathway activation. Phosphatidylinositols (PI) and their derivatives are the important secondary messenger molecule that regulates various pathways like phagocytosis, migration, endocytosis, etc. PI interacts with many actin-binding proteins and other proteins through domain interaction to activate the signaling pathway. Elucidating the direct role of dietary fatty acids in activating various pathways and type of signaling molecules affected by the PI pool is a new challenge to explore. We hypothesize that being lipid derivatives PI pool and types should show dependence on dietary fatty acids types. Since the PI levels are greatly affected in Alzheimer’s disease the therapeutic strategy could be design to normalize the PI metabolism.

## Abbreviations

PI: Phosphatidylinositol
PIP2: Phosphatidylinositol 4, 5- bisphosphate- PI 4, 5- P2
PIP3: Phosphatidylinositol 3, 4, 5- triphosphate- PI 3, 4, 5- P3
PI3K: Phosphatidylinositol-3 kinase
PI5K: Phosphatidylinositol-5 kinase
PI4P5K: Phosphatidylinositol-4- phosphate 5-kinase
PKA: Protein kinase A
PTEN: Tensin homology
PE: Phosphatidylethanolamine
PC: Phaphatidylcholine
Myo1e: Membrane-associated actin regulation factors-1e
PLC: Phospholipase C
DAG: Diacylglycerol
AD: Alzheimer’s disease
CNS: Central nervous system
Aβ: Amyloid- beta
APP: Amyloid precursor protein
NFTs: Neurofibrillary tangles
ARP2/3: Actin-related protein complex 2/3
PUFA: Polyunsaturated fatty acids
SHIP2: Src homology domain-containing inositol 5-phosphatase
*INPP5E*: Inositol 1,4,5- triphosphate- IP3, Synaptojanin1- SYJN1 5′-phosphatase inositol polyphosphate 5-phosphatase E
*MTMR*: Myotubularin related protein
DHA: Docosahexaenoic acid
EPA: Eicosapentaenoic acid
ARA: Arachidonic acid
GPR120: G-protein coupled receptor 120
RXR: Retinoid X receptor
